# Sleep Disturbances and Their Correlates in Essential Tremor and Essential Tremor Plus: A Questionnaire-Based Case–Control Study

**DOI:** 10.5334/tohm.1234

**Published:** 2026-09-03

**Authors:** Ravi Prakash Singh, S. Mythirayee, Doniparthi Venkata Seshagiri, Rohan Mohale, Pramod Kumar Pal, Bindu M. Kutty, Jitender Saini, Nitish L. Kamble, Vikram Holla, Ravi Yadav

**Affiliations:** 1 Department of Neurology, Sri Aurobindo Medical College and Post graduate institute, Indore, India; 2 Department of Neurology, National Institute of Mental Health and Neurosciences (NIMHANS), Bengaluru, India; 3 Centre for Consciousness Studies, Department of Neurophysiology, National Institute of Mental Health and Neurosciences (NIMHANS), Bengaluru, India; 4 Department of Neuroimaging and Interventional Radiology, National Institute of Mental Health and Neurosciences (NIMHANS), Bengaluru, India

**Keywords:** Essential tremor, Essential tremor plus, Sleep disturbances, Pittsburgh Sleep Quality Index, Epworth Sleepiness Scale, REM sleep behaviour disorder, Restless leg syndrome, Non-motor symptoms

## Abstract

**Background:**

Essential tremor (ET) has been increasingly recognized to involve non-motor symptoms, including sleep disturbances. Essential Tremor Plus (ET-plus), is characterized by additional neurological “soft signs” and may display greater non-motor symptom burden. Despite emerging evidence from polysomnographic studies, large-scale subjective evaluations of sleep across the ET spectrum remain limited.

**Objectives:**

To assess the prevalence and severity of sleep disturbances in ET and ET-plus using validated sleep questionnaires, and to compare with healthy controls.

**Methods:**

This study was conducted at NIMHANS, Bengaluru, and included 100 patients (60 ET and 40 ET-plus) and 100 healthy controls. Tremor severity was assessed using TETRAS and FTMRS, and sleep symptoms with ESS, PSQI, Mayo Sleep Questionnaire, RLS-Q, BQ, GAD-7 and PHQ-9. Non-parametric tests and effect size estimations were used for group comparisons.

**Results:**

ET-plus patients were older and exhibited greater tremor severity than ET. Global PSQI scores differed significantly among ET and ET Plus vs controls (p < 0.001), with ET-plus reporting the poorest sleep quality. ET and ET-plus demonstrated significantly higher ESS (p < 0.001) and greater prevalence of probable RBD, RLS, and sleep apnea risk compared with controls. Tremor disability scores correlated with PSQI scores in ET-plus, suggesting an interaction between motor and non-motor burden.

**Conclusions:**

Both ET and ET plus have significant sleep dysfunction with ET-plus patients showing markedly poorer sleep quality, higher daytime somnolence, and greater parasomnia prevalence than ET and controls. These findings highlight the clinical relevance of routine sleep assessment in ET and may impact quality of life and management.

## Introduction

Essential tremor (ET), is characterized by a persistent bilateral postural or kinetic upper limb tremor lasting at least three years. Tremor may extend to the head, voice, or lower limbs but excludes isolated head or voice tremor [1,2,3]. The presence of subtle neurological features such as mild questionable dystonia, cognitive complaints (e.g., forgetfulness), or gait imbalance along with tremor is classified under a distinct entity termed Essential Tremor Plus (ET-plus) according to the International Parkinson and Movement Disorder Society (IPMDS) tremor classification. Thus, ET-plus is differentiated from classical ET by the presence of these additional “soft” neurological signs [3]. However, the clinical significance of ET-plus remains debated. The term was introduced as a clinical classification to capture heterogeneity within the ET spectrum, but subsequent studies have questioned whether ET-plus represents a distinct subtype or a disease stage that may emerge with increasing age and disease duration [4]. Longitudinal studies have also shown that the ET plus designation is unstable over time, which further supports the possibility that some of these associated neurological features evolve during the course of ET [5]. Conversely, the presence of a distinct additional neurological features, along with its clinical heterogeneity, has kept ET plus under consideration as a clinically meaningful phenotype within the broader ET spectrum. Whether ET plus therefore represents a distinct clinical phenotype, a stage of disease evolution, or some combination of both remains an open question.

Emerging evidence suggests that ET and ET plus, much like other neurodegenerative disorders such as Parkinson’s disease, are not confined to motor abnormalities alone. In addition to tremor, patients frequently exhibit a range of non-motor symptoms including fatigue, anxiety, depression, cognitive impairment, gait and sleep disorders that collectively contribute to significant functional disability and reduced health-related quality of life [6,7,8,9]. Clinical observations suggests that ET-plus patients are typically associated with older age at onset, a higher frequency of head tremor, and are more often accompanied by non-motor features such as anxiety, depression, and probable REM sleep behavior disorder (RBD) compared with ET patients [10]. ET exhibits clinical heterogeneity, with a subset of patients demonstrating additional neurological soft signs, commonly categorized under ET-plus. This framework was introduced to better characterize clinical heterogeneity within the ET spectrum; however, whether ET-plus represents a distinct clinical subtype or a stage of disease remains unresolved.

Sleep disturbances, well recognized in Parkinson’s disease and other synucleinopathies, have also been reported in ET and ET plus [9,11]. As ET is increasingly viewed as a neurodegenerative disorder, disruptions in sleep may reflect involvement of sleep-regulating structures such as the locus coeruleus. Post-mortem studies by Louis et al showing increased brainstem Lewy bodies in ET further support a possible neurobiological link between ET and sleep abnormalities [12].

Sleep disturbances in ET have been reported both by questionnaire survey and polysomnographic studies [6,8,11,13]. Patients typically report poor subjective sleep quality, excessive daytime somnolence, symptoms of insomnia, and elevated occurrence of probable RBD compared to control participants [14,15,16,17,18]. Questionnaire-based studies have suggested that these sleep disturbances may be associated with increased severities of tremor and mood-related symptoms although these results have been variable and limited by small numbers and differences in study design [15,18,19]. Previous studies suggest that sleep disturbances are more common in patients with ET; however, relatively few studies have objectively evaluated these abnormalities using polysomnography (PSG) [11]. The large-scale assessment of PSG requires more practical and methodological solutions which currently prevent researchers from executing their studies. The process of participant recruitment for overnight studies becomes challenging because people do not want to take part in comprehensive laboratory tests which decreases study participation and creates smaller participant groups. The PSG recording process needs trained sleep technologists or clinicians or researchers to score data through exact manual methods which follow the American Academy of Sleep Medicine standards.

In our cohort, PSG studies have provided important insights into sleep abnormalities associated with ET and ET-plus [11]. Compared with healthy controls, ET and ET plus patients demonstrate alterations in sleep macroarchitecture characterized by reduced total sleep time, decreased sleep efficiency, prolonged REM latency, and increased wake after sleep onset. ET and ET Plus patients also exhibit reductions in N2 and slow-wave sleep (N3), suggesting impaired ability to maintain restorative sleep stages [11]. In addition, elevated apnea–hypopnea indices, higher snore indices, increased arousal indices, and periodic limb movement activity have been reported [11]. Most previous studies have approached ET as a relatively uniform clinical entity, and there remains a paucity of data systematically evaluating non-motor features, particularly sleep disturbances, across these subgroups. In this context, comparing sleep disturbances between ET and ET-plus may help delineate differences in clinical burden and further refine our understanding of the spectrum of the disorder. The present study aimed to evaluate sleep disturbances and associated non-motor symptoms in a large cohort of patients with ET and ET-plus, and to compare their sleep profiles with those of healthy controls using validated sleep questionnaires.

## Methodology

This was a cross-sectional case-control study. Participants were recruited over a two-year period from the Neurology and Movement Disorder outpatient clinics at National Institute of Mental Health and Neuro Sciences, Bangaluru, India. Inclusion criteria included adults of both genders diagnosed with ET or ET-plus according to the IPMDS consensus on tremor classification [3]. Sample size was determined based on feasibility and availability of eligible participants during the recruitment period. A total of 100 patients (60 ET, 40 ET-plus) and 100 sex- and age-matched healthy control participants without neurological/major psychiatric disease were enrolled in the study. Healthy controls were recruited from accompanying relatives and community volunteers. Participants with other notable movement disorders, secondary tremor syndromes, untreated major psychiatric disorders, and life-threatening systemic diseases were excluded from participation in this study. The study protocol was approved by the Institutional Ethics Committee (IEC Number-NO.NIMH/DO/IEC)(BS & NS Division)/2021–23, dated 16.11.2022). Written informed consent was obtained from all participants prior to study participation.

### Clinical assessment

Comprehensive demographic and clinical information was gathered for all participants, including age, sex, age at tremor onset, disease duration, alcohol responsiveness of tremor, comorbid conditions, and family history of ET. A detailed medical history was obtained to identify comorbid conditions such as hypertension, diabetes mellitus, ischemic heart disease, and hypothyroidism, as well as the use of sympathomimetic medications that could potentially influence tremor. All participants underwent a comprehensive neurological examination, and operational classification into ET or ET-plus was performed according to the International Parkinson and Movement Disorder Society (IPMDS) consensus criteria [3]. Tremor phenomenology was carefully assessed, including the presence and distribution of tremor in the upper limbs, lower limbs, head, voice, and tongue. Tremor was evaluated in different states, including resting, postural, simple kinetic, and intentional tremor [3]. Particular attention was paid to identifying the additional neurological signs required for ET-plus classification according to the IPMDS consensus criteria [3]. These included questionable dystonic posturing, impaired tandem gait, bradykinesia, rigidity, cerebellar signs (ataxia) and cognitive complaints such as forgetfulness. Given the lack of standardized definitions for some soft signs, previously described clinical features were used to identify questionable dystonia, including “spooning” (wrist flexion with metacarpophalangeal hyperextension) [20] and Index Finger Pointing (IFP), defined as extension of the index finger with partial or full flexion of the remaining digits, particularly during walking [21,22,23]. Tandem gait was assessed using 10 consecutive heel-to-toe steps and was considered abnormal if two or more steps deviated from the line, with reproducibility required on two attempts [24]. Cognitive complaints were assessed using clinical history, supported by Mini-Mental State Examination (MMSE) scores where appropriate. Mild cognitive impairment was defined as MMSE <17 in illiterate individuals and <24 in individuals with middle-school education or higher, particularly when associated with reduced recall scores [15,25,26]. Rest tremor was evaluated with participants standing with relaxed arms by their sides or seated with hands resting on the lap with forearms supported against gravity [15,27]. Any discrepancies in clinical assessment between examiners were resolved through consensus discussions.

Tremor severity and functional impact were assessed using the Essential Tremor Rating Assessment Scale (TETRAS) and the Fahn–Tolosa–Marin Tremor Rating Scale (FTMRS). The TETRAS includes an activities of daily living (ADL) subscale and a performance subscale evaluating tremor distribution and amplitude on a 0–4 scale [28]. The FTMRS comprises Part A (tremor severity), Part B (motor tasks including handwriting, drawing, and pouring), and Part C (functional disability), with the total score representing the sum of all components [29].

### Sleep Assesssment and Questionnaire

Sleep disturbances were assessed using validated questionnaires covering multiple domains, including sleep quality, latency, duration, efficiency, excessive daytime sleepiness, RLS, probable RBD and other parasomnias. The Pittsburgh Sleep Quality Index (PSQI) is a 19-item self-reported questionnaire that assesses sleep quality over the previous one month and consists of 19 items grouped into seven components: subjective sleep quality, sleep latency, sleep duration, habitual sleep efficiency, sleep disturbances, use of sleep medication, and daytime dysfunction. Each component is scored from 0 to 3, yielding a global score ranging from 0 to 21, where a score ≥5 indicates poor sleep quality [30]. The Epworth Sleepiness Scale is an 8-item self-report scale used to assess daytime sleepiness. Respondents rate their likelihood of dozing in common daily situations on a scale of 0 to 3, with total scores ranging from 0 to 24; higher scores reflect greater daytime somnolence and a score of >10 was taken as severe excessive daytime dysfunction [31]. The Mayo Sleep Questionnaire–Informant Version (MSQ-I) was used which is a validated informant-based screening questionnaire used to identify probable REM sleep behaviour disorder (pRBD), restless legs syndrome (RLS), and sleepwalking (somnambulism) [32,33]. Question 1 screens for probable RBD based on a history of dream enactment behaviour. Question 3 screens for RLS, where a positive response suggests leg restlessness interfering with sleep. Question 4 screens for sleepwalking, with a “yes” response indicating possible somnambulism. The questionnaire has demonstrated good sensitivity and specificity for identifying probable RBD in community-based populations [33]. The Berlin Questionnaire is a screening tool for OSA risk and assesses three domains: snoring behaviour, daytime sleepiness, and the presence of hypertension or elevated body mass index. A positive score in at least two categories indicates a high risk for OSA [34]. The International Restless Legs Syndrome Rating Scale (IRLS) is a 10-item Likert-based questionnaire used to assess the severity and impact of RLS symptoms. Total scores range from 0 to 40, with higher scores indicating greater severity and functional impact [35,36].

### Assessment scale for anxiety

The Generalized Anxiety Disorder-7 (GAD-7) is a brief self-administered questionnaire used to screen for and assess the severity of anxiety symptoms. It consists of 7 items scored from 0 to 3, with total scores ranging from 0 to 21. Scores of 5, 10, and 15 indicate mild, moderate, and severe anxiety, respectively [37,38].

### Assessment scale for depression

The Patient Health Questionnaire-9 (PHQ-9) is a brief self-administered tool used to screen for and assess the severity of depressive symptoms. It is based on DSM criteria and is widely used in both clinical practice and research settings [39].

### Statistical analysis

All analyses were performed using R version 4.4.2 (R Core Team, Vienna, Austria). The distribution of continuous variables were assessed using the Shapiro–Wilk test, which confirmed that the majority of data were non-normally distributed (skewed) except for age in the control, ET and ET Plus group. Therefore, continuous and ordinal data are presented as median (interquartile range, IQR), for age (mean ± standard deviation) while categorical data are summarized as frequencies and percentages.

Group comparisons of categorical variables were conducted using the chi-square (χ²) test. For skewed continuous and ordinal data involving three groups (Control, ET, ET-plus), the Kruskal–Wallis H test was used. When the Kruskal–Wallis test indicated significant differences, Dunn’s post-hoc test with Bonferroni correction was applied for pairwise group comparisons. Direct comparisons of tremor severity between ET and ET-plus (two independent groups) were performed using the Mann–Whitney U test due to non-normal distribution of the data.

Effect sizes for non-parametric tests were reported using epsilon-squared (ɛ²) and interpreted as negligible (<0.01), small (0.01–0.06), moderate (0.06–0.14), and large (>0.14). Additional analyses were performed to examine the effect of disease duration on sleep disturbances. Patients were stratified into shorter and longer disease duration groups based on a median split (8 years), and comparisons of sleep variables were conducted between these groups. Furthermore, multivariable regression analyses were performed to evaluate differences between ET and ET-plus while adjusting for disease duration and age as a potential confounder for sleep quality. A two-tailed p-value < 0.05 was considered statistically significant.

## Results

### Demographic and Clinical Characteristics

A total of 200 participants were included. Hundred controls, 60 with ET, and 40 with ET-plus. As shown in Table 1, ET-plus patients were older compared to ET and controls. The median disease duration was 7 years (IQR: 5–12 years) in the ET group and 10 years (IQR: 6–18 years) in the ET-plus group.

**Table 1:** Demographic and clinical characteristics of study participants.


SOCIO-DEMOGRAPHICS					

CHARACTERISTIC	CONTROL (n = 100)	ET (n = 60)	ET-PLUS (n = 40)	χ² (df = 1); KRUSKAL WALLIS	p-VALUE

Age (years), mean ± SD (range)	44.0 ± 12.6 (24–69)	42.3 ± 14.5 (17–66)	54.0 ± 13.8 (22–75)	H(2) = 7.69, p = 0.021	Control vs ET: p = 1.000 (ns) Control vs ET-plus: p = 0.024 (significant) ET vs ET-plus: p = 0.058

Sex (M/F)	66/34	47/13	29/11	χ² = 2.83	p = 0.244

Disease Duration (years)		7 (IQR: 5–12)	10 (IQR: 6–18)		

Hypertension, n(%)	11 (11%)	14 (23%)	15 (38%)	1.701878	0.192043

Diabetes, n (%)	3 (3%)	6 (10%)	10 (25%)	2.979291	0.084336

Ischemic Heart Disease, n (%)	0 (0%)	6 (10%)	1 (3%)	1.081669	0.298324

Hypothyroidism, n(%)	4 (4%)	2 (3%)	1 (3%)	–	–

Tremor Feature					

Bilateral upper limb tremors	–	60 (100%)	40 (100%)	–	–

Lower limb tremors	–	14 (23%)	15 (38%)	1.7	0.192

Voice tremors	–	20 (33%)	23 (58%)	4.78	0.029*

Head tremors	–	8 (13%)	13 (33%)	4.22	0.040*

Tongue tremors	–	13 (22%)	16 (40%)	3.08	0.079


### Tremor Characteristics

The characteristics of the tremor in ET-plus and ET are listed in Table 1. All the patients had bilateral upper limb tremor. Chi-square test showed significantly increased occurrence of voice tremors (58% vs. 33%, p = 0.029) and head tremors (33% vs. 13%, p = 0.040) in ET-plus as compared to ET. Lower limb as well as tongue tremors occurred more frequently in ET-plus but the difference was not significant. The distribution of the additional soft neurological signs that qualified patients for ET-plus classification is summarized in Supplementary Table 1.

### Tremor Rating Scales

**Figure 1:**
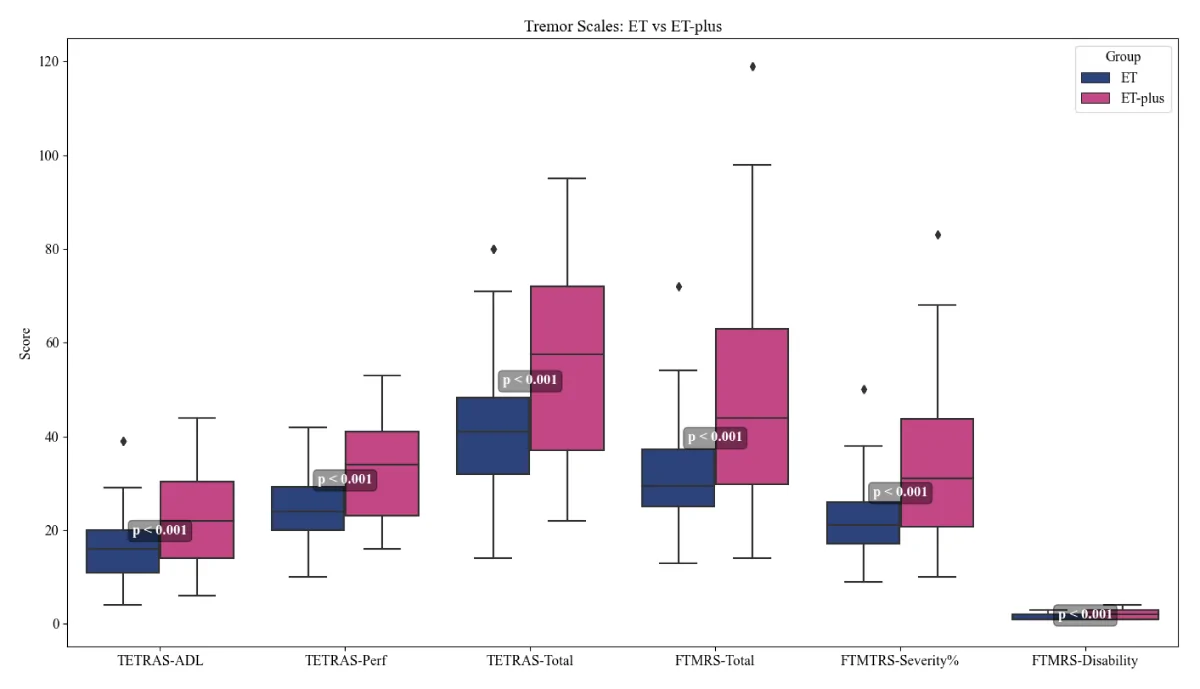
Tremor rating scales in ET and ET-plus groups.

Because tremor scores were skewed, group differences between ET and ET-plus were analysed using the Mann–Whitney U test. ET-plus patients had significantly higher scores across all TETRAS and FTMRS domains (Table 2), with large effect sizes (ɛ² = 0.74–0.88). This reflects significant differences in tremor severity and disability. The distributions are visualized in Figure 1.

**Table 2:** Tremor rating scale scores in ET vs ET-plus patients.


TREMOR SCALE	ET (n = 60)	ET-PLUS (n = 40)	MANN-WHITNEY U p-VALUE	ɛ²

TETRAS ADL subscale (0–48)	16 (11, 20)	22 (14, 30)	<0.001	0.74

TETRAS Performance subscale (0–64)	24 (20, 29)	34 (23, 41)	<0.001	0.766

TETRAS Total score (0–112)	41 (32, 48)	58 (37, 72)	<0.001	0.78

FTMRS Total score (0–144)	30 (25, 37)	44 (30, 63)	<0.001	0.798

FTMTRS Severity%(0–144)	21 (17, 26)	31 (21, 44)	<0.001	0.795

FTMRS Disability level	1 (1, 2)	2 (1, 3)	<0.001	0.884


*Data are presented as median (IQR) unless otherwise specified.

### Sleep Quality

**Figure 2:**
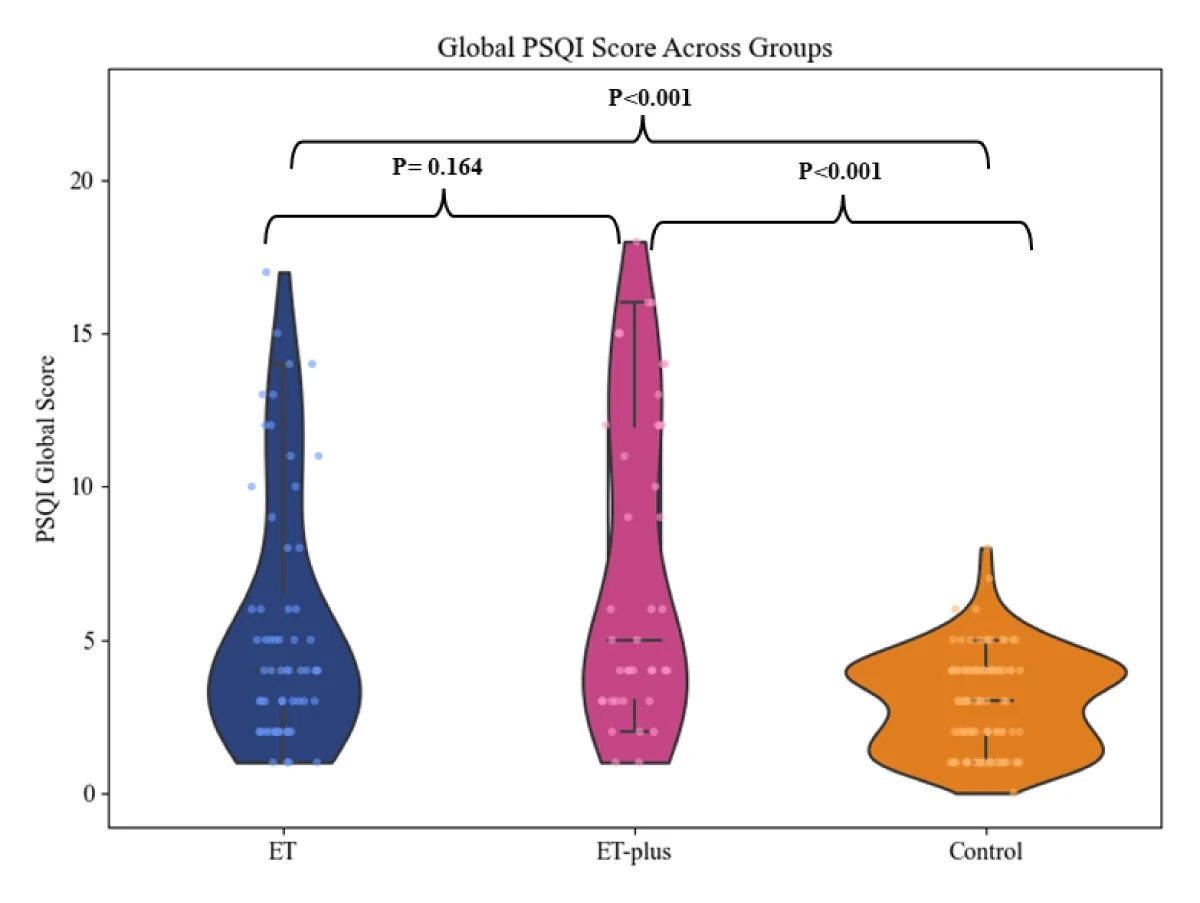
Distribution of Global PSQI scores across Control, ET, and ET-plus groups.

The Kruskal-Wallis test showed significant differences in global PSQI scores among the three groups (χ² = 30.1, p < 0.001, ɛ² = 0.151) as demonstrated in table 3. Post-hoc Dunn’s test revealed that both the ET and ET-plus patients had significantly higher PSQI scores when compared to the healthy group (both p < 0.001). There was no significant difference in the global PSQI score between the two patient groups (ET and ET-plus) (p = 0.164). In terms of individual PSQI sub components, subjective sleep quality (Component 1), sleep disturbance (Component 5), sleep medication (Component 6) and sleep efficiency (Component 4) were significantly poorer in both ET and ET-plus than controls. However, there was no significant difference between ET and ET-plus groups for all these components. The distribution of global PSQI is illustrated in Figure 2. Patients were stratified into shorter and longer disease duration groups based on the median duration. The mean global PSQI score was 6.31 ± 4.68 in the shorter duration group and 6.02 ± 4.40 in the longer duration group. There was no statistically significant difference in sleep quality between the two groups. Multivariable regression analysis was performed to evaluate the association between ET-plus status and global PSQI score after adjusting for disease duration. ET-plus was found to be significantly associated with poorer PSQI scores (β = 1.85, p = 0.045), whereas disease duration was not significantly associated with PSQI scores (β = 0.07, p = 0.31).

**Table 3:** Pittsburgh Sleep Quality Index (PSQI) scores across groups.


PSQI COMPONENTS	CONTROL (n = 100)	ET (n = 60)	ET-PLUS (n = 40)	KRUSKAL-WALLIS (df = 2)	p-VALUE	ɛ²	ET vs CONTROL	ET-PLUS vs CONTROL	ET vs ET-PLUS

Global PSQI score	3 (2, 4)	4 (3, 7)	5 (3, 12)	30.1	<0.001	0.151	<0.001*	<0.001*	0.164

Component 1: Subjective sleep quality	1 (0, 1)	1 (1, 2)	1 (1, 2)	48.91	<0.001	0.246	<0.001*	<0.001*	0.095

Component 2: Sleep latency	1 (1, 1)	1 (0, 1)	1 (0, 1)	3.51	0.173	0.018	0.139	0.909	0.561

Component 3: Sleep duration	0 (0, 0)	0 (0, 1)	1 (0, 1)	14.82	<0.001	0.075	0.186	<0.001*	0.128

Component 4: Sleep efficiency	0 (0, 1)	1 (0, 1)	1 (0, 2)	11.94	0.003	0.06	0.018*	0.010*	0.664

Component 5: Sleep disturbances	1 (0, 1)	1 (1, 1)	1 (1, 2)	38.98	<0.001	0.196	<0.001*	<0.001*	0.333

Component 6: Sleep medication use	0 (0, 0)	0 (0, 0)	0 (0, 0)	21.37	<0.001	0.107	<0.001*	<0.001*	0.577

Component 7: Daytime dysfunction	0 (0, 1)	1 (0, 1)	1 (1, 2)	37.91	<0.001	0.191	<0.001*	<0.001*	0.039*


*Data are presented as median (IQR) unless otherwise specified.

### Probable RBD, RLS and Sleep-Disordered Breathing

**Figure 3:**
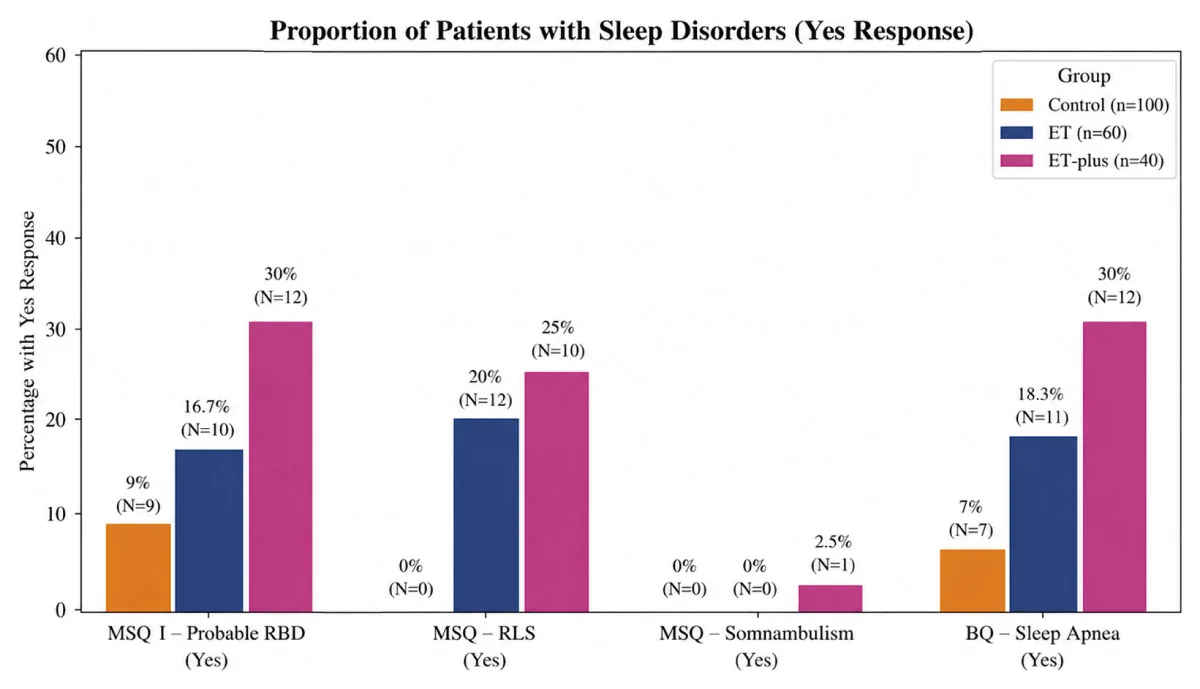
Prevalence of other sleep disorders across ET, ET plus and Controls.

Questionnaire-based screening showed a significantly higher prevalence of probable RBD in both ET (17%) and ET-plus (30%) patients compared with controls (9%), with no significant difference between the ET and ET-plus groups. Similarly, RLS was significantly more prevalent in both ET (20%) and ET-plus (25%) than in controls (0%), whereas a high risk for obstructive sleep apnea was significantly higher only in the ET-plus group (30%) compared with controls (7%). The prevalence of these questionnaire-based sleep disorders is summarized in Table 4 and illustrated in Figure 3.

**Table 4:** Probable RBD, RLS, Somnambulism and Sleep Apnea screening across groups.


VARIABLE	CONTROL (n = 100)	ET (n = 60)	ET-PLUS (n = 40)	χ² (df = 2)	p-VALUE	ɛ²	ET vs CONTROL	ET-PLUS vs CONTROL	ET vs ET-PLUS

MSQ I – Probable RBD (yes/no)	9%	17%	30%	14.94	<0.001	0.075	0.012*	<0.001*	0.639

MSQ – RLS (yes/no)	0%	20%	25%	37.32	<0.001	0.188	<0.001*	<0.001*	0.133

MSQ – Somnambulism (yes/no)	0%	0%	3%	4	0.135	0.02	–	0.254	0.439

BQ – Sleep Apnea (high risk)	7%	18%	30%	12.54	0.002	0.063	0.073	0.001*	0.367


### Cognitive, Sleepiness, and Psychiatric Measures

The Kruskal–Wallis test showed significant differences between groups for excessive daytime sleepiness (ESS, p < 0.001), RLS severity (p < 0.001), anxiety severity (GAD-7, p < 0.001), and depression severity (PHQ-9, p < 0.001) (Table 5). Post-hoc analysis demonstrated that both the ET and ET-plus groups had significantly higher ESS, RLS, GAD-7, and PHQ-9 scores compared with controls. However, no statistically significant differences were observed between the ET and ET-plus groups for these measures. Anxiety showed a large effect size, depression showed a moderate-to-large effect size, and excessive daytime sleepiness showed a moderate effect size. Although MMSE scores were statistically lower in the ET-plus group (p < 0.001), the median difference was small (29 vs. 30), suggesting that the statistical difference should be interpreted cautiously as its clinical significance is uncertain and may have been influenced by a small number of lower MMSE values.

**Table 5:** Cognitive, sleepiness, and psychiatric measures across groups.


VARIABLE	CONTROL (n = 100)	ET (n = 60)	ET-PLUS (n = 40)	KRUSKAL WALLIS (df = 2)	p-VALUE	ɛ²	ET vs CONTROL	ET-PLUS vs CONTROL	ET vs ET-PLUS

MMSE score	30 (29, 30)	30 (30, 30)	29 (23, 30)	–	–	–	–	–	<0.001*

ESS > 9 (Excessive sleepiness)	1 (0, 3)	3 (1, 5)	4 (3, 7)	21.15	<0.001	0.106	0.007*	<0.001*	0.301

RLS severity (0–40)	0 (0, 0)	6 (5, 12)	5 (3, 9)	26.86	<0.001	0.135	<0.001*	<0.001*	0.795

GAD-7 (Anxiety severity)	0 (0, 3)	6 (3, 9)	5 (4, 7)	83.02	<0.001	0.419	<0.001*	<0.001*	0.974

PHQ-9 (Depression severity)	3 (1, 6)	5 (3, 9)	5 (3, 6)	23.48	<0.001	0.118	<0.001*	0.006*	0.665


*Data are presented as median (IQR) unless otherwise specified.

### Correlation Analyses

**Figure 4:**
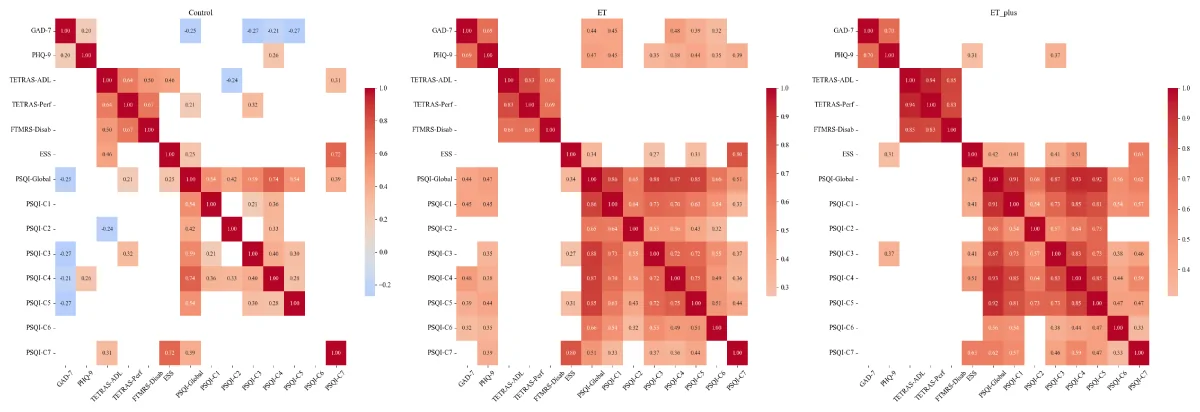
Correlation matrices of clinical and sleep parameters across ET, ET plus and Controls.

The correlation matrices (Figure 4) demonstrated stronger and more extensive relationships among tremor severity, sleep quality, daytime sleepiness, and psychiatric measures in both the ET and ET-plus groups compared with controls. In the ET group, global sleep quality (PSQI-Global) showed significant positive correlations with excessive daytime sleepiness (ESS; *r* = 0.34), TETRAS-ADL (*r* = 0.34), and TETRAS-Performance (*r* = 0.34). Similar findings were observed in the ET-plus group, where global PSQI correlated with ESS (*r* = 0.42), TETRAS-ADL (*r* = 0.42), and TETRAS-Performance (*r* = 0.41), suggesting an association between poorer sleep quality, greater daytime sleepiness, and increased tremor severity.

Global PSQI demonstrated strong positive correlations with its individual component scores in both ET and ET-plus groups, indicating that impairment in overall sleep quality was accompanied by abnormalities across multiple sleep domains. Anxiety and depression scores were strongly correlated with each other in both ET (*r* = 0.69) and ET-plus (*r* = 0.70). In addition, both GAD-7 and PHQ-9 scores showed moderate positive correlations with global PSQI in ET (GAD-7: *r* = 0.44; PHQ-9: *r* = 0.47) and ET-plus (GAD-7: *r* = 0.42; PHQ-9: *r* = 0.41), highlighting the close relationship between mood symptoms and poor sleep quality. ESS also demonstrated a strong positive correlation with the PSQI daytime dysfunction component in both ET (*r* = 0.80) and ET-plus (*r* = 0.63). In contrast, the control group exhibited fewer and weaker correlations among sleep, psychiatric, and clinical measures.

Linear regression analysis was conducted to determine the difference in global PSQI score between ET and ET-plus with adjustments for age and disease duration. In addition, ET-plus remained not a predictor of the global PSQI score even after adjustment (β = 0.93, 95% CI –1.01 to 2.88, p = 0.344). Age was found to be independently associated with global PSQI scores (β = 0.08 per year, 95% CI 0.02–0.15, p = 0.014), but disease duration had no association with sleep quality (β = 0.02, 95% CI –0.11 to 0.15, p = 0.762). The regression model as a whole had statistical significance (R² = 0.108, adjusted R² = 0.080; F(3,96) = 3, p = 0.012).

## Discussion

The present study comprehensively assessed subjective sleep disturbances in patients with ET and ET-plus using validated questionnaires and compared them with healthy controls. Our findings demonstrate that sleep abnormalities are common across the ET and ET-plus spectrum, with both groups showing greater sleep-related burden than healthy controls, while differences between ET and ET-plus were not consistently statistically significant. A systematic review by Jiménez-Jiménez et al. further supports these observations, emphasizing the growing recognition of diverse sleep disturbances in ET spectrum and their clinical relevance [9].

### Clinical and Demographic Characteristics

In our cohort, the age at tremor onset ranged from 17 to 75 years, demonstrating a bimodal distribution similar to that reported in the meta-analysis by Song et al. [40]. Among the 100 patients, 60% were classified as ET and 40% as ET-plus, a distribution differing from the Chinese survey by Sun et al., which reported 47.1% ET, [15] but comparable to the findings of Lolekha et al., who observed 52.9% ET [41] Across all groups, a mild male predominance was observed, with male-to-female ratios of 66:34 in controls, 47:13 in the ET group, and 29:11 in the ET-plus group. Similarly, the meta-analysis by Louis et al. did not identify a significant sex difference in the prevalence of ET [42]. In contrast, Sun et al. reported that females were more prevalent in the ET-plus group compared with the pure ET group [15]. In our cohort, ET-plus patients were older than ET patients, though the difference was not significant. This observation is consistent with previous reports showing that ET-plus patients are generally older than those with ET, although whether this reflects a distinct clinical phenotype or differences in disease evolution remains to be established [15].

In our study, cranial tremors were more frequent in the ET-plus group, with both head and voice tremor occurring more often than in ET. This pattern is consistent with prior literature, which also highlights greater cranial involvement in ET-plus compared with ET [25,41].

We also identified lower limb tremor in 38% of ET-plus and 23% of ET patients-figures somewhat higher than earlier reports suggesting a prevalence of 10–30% [2,43,44]. Detecting such subtle lower extremity tremors can be challenging, as emphasized by prior studies citing poor interrater reliability in their recognition [28].

In line with prior research, ET-plus patients in our cohort demonstrated more severe tremor and greater functional impairment compared to ET. This is consistent with Bellows et al., who reported significantly higher TETRAS scores and performance impairment in ET-plus [25]. Similarly, Peng et al. noted consistently elevated tremor severity scores in ET-plus during clinical evaluations [45]. Moreover, a recent nationwide study from China by Sun Q et al. reported that patients with late-onset ET had significantly higher TETRAS-I and TETRAS-II scores than those with early-onset ET (all *P* < 0.001). The study also found that late-onset ET was more frequently classified as ET-plus, suggesting an association between later disease onset, greater tremor severity, and ET-plus classification [15]. The relationship between ET and ET-plus remains an area of ongoing debate. Although ET-plus has been proposed by some investigators to represent a later stage of disease evolution, particularly in relation to increasing age and disease duration, the additional neurological features defining ET-plus may also represent clinically meaningful phenotypic heterogeneity. In our cohort, ET-plus was associated with greater tremor severity and functional impairment, whereas differences in sleep and other non-motor measures between ET and ET-plus were not consistently statistically significant. Thus, our findings support the clinical relevance of examining ET-plus as a distinct phenotype while acknowledging that cross-sectional data cannot determine whether these features represent disease evolution, phenotypic heterogeneity, or distinct underlying pathobiology.

### Sleep Quality and Subjective Sleep Disturbances

This study demonstrates that patients with ET and ET-plus experience greater sleep disturbances than healthy controls. Overall sleep quality was poorer in both ET and ET-plus groups compared with healthy controls. Although ET-plus showed numerically greater impairment than ET, the difference between the two patient groups was not statistically significant. The median global PSQI score was numerically highest in ET-plus compared to ET and controls. Component-wise, ET-plus patients reported worse subjective sleep quality and shorter sleep duration than controls, whereas sleep latency did not differ between groups. Reduced sleep efficiency (Component 4) and shorter sleep duration (Component 3) highlighted that ET-plus patients experience more fragmented and less restorative sleep. Nearly two-thirds of ET-plus and half of ET patients exceeded the threshold for poor sleep quality (PSQI > 5), compared with only 20% of controls. These results are consistent with previous reports by Chandran et al. and Jiménez-Jiménez et al., and who found poor subjective sleep quality and increased sleep latency among ET patients relative to controls [6,9].

Prior studies by various authors also demonstrated higher PSQI scores in ET, suggesting that disrupted sleep may be a core non-motor manifestation rather than a comorbidity [16,46,47,48,49].

We observed significantly greater daytime sleepiness in both ET and ET-plus compared with controls. Although ET-plus had numerically higher ESS scores than ET, the difference between the two groups was not statistically significant. The prior studies have also revealed that daytime sleepiness is a common and clinically significant manifestation in patients with ET and ET plus [6,8,14,46,50]. It may be attributed to sleep fragmentation at night, side effects of drugs like beta blockers and primidone, or changes in circadian rhythm [51,52]. This worsens quality of life in the form of increased fatigue, poor cognitive functions, and difficulty performing routine activities.

### Probable RBD and RLS

One important observation was the higher occurrence of questionnaire-identified probable RBD and RLS in both ET and ET-plus patients compared with controls. Although the prevalence was numerically higher in ET-plus than in ET (probable RBD: 30% vs. 17%; RLS: 25% vs. 20%), these differences were not statistically significant. This corroborates the findings from PSG studies by Salsone et al., and Bugalho et al., who have observed lack of atonia during REM sleep in ET patients and PSG-diagnosed RBD [13,53]. This is consistent with previous reports as indicated by Jimenez et al. and Lacerte et al., that RLS occurs twice as often in ET patients than in the normal population(9,19). In our previous PSG study involving a subset of the present cohort, we also observed a higher frequency of probable RBD and RLS in ET and ET-plus patients than in control [11]. The increased frequency of questionnaire-identified probable RBD in ET and ET-plus patients, although less frequent than in Parkinson’s disease, raises the possibility of shared neurodegenerative pathways involving brainstem structures such as the sublaterodorsal nucleus and pontine tegmentum [54,55]. However, because probable RBD was identified using a screening questionnaire rather than PSG, these findings should be interpreted cautiously and require confirmation in larger PSG-based studies.

The increased frequency of RLS and probable RBD may reflect involvement of dopaminergic and brainstem circuits, supporting the concept that ET is associated with abnormalities extending beyond motor pathways [13].

### Mood, Cognition, and Sleep Interactions

The patients in our cohort exhibited significantly higher anxiety (GAD-7) and depression (PHQ-9) scores than healthy controls, consistent with previous studies [4,55]. Our study demonstrated significant positive correlations between anxiety, depression, and global PSQI scores, indicating a bidirectional link between mood and sleep where one may affect the other. Furthermore, ET-plus patients had slightly lower MMSE scores than ET patients (median 29 vs. 30). Although this difference reached statistical significance, the absolute difference was small and may not be clinically meaningful in the present cohort, particularly given the relatively small sample size. Similar observations have been reported previously [15].

### Pathophysiological Considerations

The mechanisms underlying poor sleep in ET and ET Plus patients likely involve both psychological and neurobiological factors. Neurodegeneration involving brainstem and cerebellothalamic circuits, particularly locus coeruleus and red nucleus, may contribute to dysregulation of sleep. Dysregulation of GABAergic, dopaminergic, and noradrenergic systems could underpin these findings. The presence of questionnaire-identified probable RBD and RLS in both ET and ET-plus patients in our study further supports the possibility of a neurodegenerative continuum. Postmortem studies have shown increased Lewy body deposition in the locus coeruleus of ET patients implicating noradrenergic dysfunction in sleep-wake control [12]. Such findings support the hypothesis that sleep abnormalities in ET may share mechanisms overlapping with synucleinopathies such as Parkinson’s disease. However, recent pathological studies, including the the work of Louis et al demonstrated no meaningful differences in cerebellar pathology between ET and ET-plus, questioning whether they represent distinct pathological entities [56]. Future studies should integrate clinical characteristics with imaging, physiological markers, and genetic profiles to clarify whether ET-plus reflects disease progression, compensatory mechanisms, or distinct underlying pathobiological processes within the broader ET spectrum.

### Clinical Implications

The consistent finding of sleep disturbances in both ET and ET-plus-underscores the need for routine sleep assessment in clinical practice. Poor sleep not only worsens tremor and fatigue but also increases anxiety and depression, creating a feedback loop that reduces quality of life. Questionnaires like PSQI, ESS, and IRLS-Q provide efficient and scalable screening tools, especially in settings where PSG is not feasible. Identification and management of sleep disturbances in these patients through behavioural interventions, pharmacotherapy, or referral to sleep clinics could possibly improve patient outcomes.

### Strengths and Limitations and Future Directions

A notable strength of our study is the inclusion of a large, well-characterized native Indian cohort, which provides meaningful data relevant to asian population. The use of age- and sex-matched healthy controls and standardized multi-domain clinical and sleep questionnaires adds to the robustness of the findings. However, the cross-sectional design limits causal interpretation, and questionnaire-based measures may not fully capture objective sleep architecture. Our study relied on validated questionnaire-based screening tools to identify sleep disorders. Consequently, diagnoses of conditions such as RBD, OSA, and RLS should be interpreted as probable rather than definitive, as confirmation with overnight video PSG was not performed. Though, these questionnaires have demonstrated good diagnostic performance in prior studies, errors in classification remains possible. Despite the lack of statistical differences in sex distribution, it has been documented that sex affects the quality of sleep. The possibility of residual confounding related to demographic differences remains, despite the adjustment for age in multivariable analysis. Future studies incorporating longitudinal follow-up and polysomnographic assessment, along with biomarkers and neuroimaging, may help clarify underlying mechanisms linking sleep disturbances, mood symptoms, and disease progression in ET and ET-plus.

## Conclusion

Sleep abnormalities were common in ET and ET-plus patients, suggesting that sleep dysfunction is an important non-motor feature of these disorders. Both ET and ET-plus patients demonstrated significantly greater sleep disturbances than healthy controls. Although ET-plus generally showed numerically greater impairment than ET, these differences were not consistently statistically significant. These findings support the concept that sleep abnormalities are part of the broader clinical spectrum of ET and ET-plus. Further studies incorporating objective sleep assessments are needed to determine whether the numerically greater impairment observed in ET-plus reflects underlying biological differences.

## Additional File

The additional file for this article can be found as follows:

10.5334/tohm.1234.s1 Supplemental File 1. Frequency of additional soft neurological signs in ET plus cases.
