# Supplementary material for: Sleep Disturbances and Their Correlates in Essential Tremor and Essential Tremor Plus: A Questionnaire-Based Case–Control Study
Source: Tremor Other Hyperkinet Mov (N Y). 2026 Sep 3;16:50. doi: 10.5334/tohm.1234 (PMC13544043; doi:10.5334/tohm.1234)
Supplement: Supplementary file 1 [file tohm-16-1-1234-s1.pdf]

Supplementary Table 1 : Frequency of additional soft neurological signs in ET plus cases

Soft neurological signs	Number (N = 40)	Percentage %
**Rest Tremor**	19	47.5%
**Tandem gait impairment**	17	42.5%
**Forgetfulness**	16	40.0%
**Bradykinesia**	1	2.5%
**Rigidity**	0	0%
**Dystonic Posturing**	15	37.50%
**Myoclonus**	0	%
**Gaze restriction**	0	0%
**Oculomotor abnormalities**	0	0%
**Primitive reflexes**	0	0%
**Ataxia**	3	7.5%
